# Characterization of Distributed Microfabricated Strain Gauges on Stretchable Sensor Networks for Structural Applications

**DOI:** 10.3390/s18103260

**Published:** 2018-09-28

**Authors:** Xiyuan Chen, Tanay Topac, Wyatt Smith, Purim Ladpli, Cheng Liu, Fu-Kuo Chang

**Affiliations:** 1 Department of Mechanical Engineering, Stanford University, Building 530, 440 Escondido Mall, Stanford, CA 94305, USA; wcsmith@stanford.edu (W.S.); chengliu@stanford.edu (C.L.); 2 Department of Aeronautics and Astronautics, Stanford University, Durand Building, 496 Lomita Mall, Stanford, CA 94305, USA; tanaytopac@stanford.edu (T.T.); pladpli@stanford.edu (P.L.); fkchang@stanford.edu (F.-K.C.)

**Keywords:** structural health monitoring, strain gauge, sensor network, distributed strain measurement, smart structure

## Abstract

Smart structures mimic biological systems by using thousands of sensors serving as a nervous system analog. One approach to give structures this sensing ability is to develop a multifunctional sensor network. Previous work has demonstrated stretchable sensor networks consisting of temperature sensors and impact detectors for monitoring external environments and interacting with other objects. The objective of this work is to develop distributed, robust and reliable strain gauges for obtaining the strain distribution of a designated region on the target structure. Here, we report a stretchable network that has 27 rosette strain gauges, 6 resistive temperature devices and 8 piezoelectric transducers symmetrically distributed over an area of 150 × 150 mm to map and quantify multiple physical stimuli with a spatial resolution of 2.5 × 2.5 mm. We performed computational modeling of the network stretching process to improve measurement accuracy and conducted experimental characterizations of the microfabricated strain gauges to verify their gauge factor and temperature coefficient. Collectively, the results represent a robust and reliable sensing system that is able to generate a distributed strain profile of a common structure. The reported strain gauge network may find a wide range of applications in morphing wings, smart buildings, autonomous cars and intelligent robots.

## 1. Introduction

Smart structures are designed to sense their environmental changes and respond adaptively, whereas conventional structures can only provide strength and carry loads. A smart system is composed of multiple smart structures plus a command-and-control unit which is able to describe and analyze a situation, distinguish between normal load conditions and abnormal load cases and make decisions in a predictive or accommodative manner. The concept of smart systems has been extensively used in a wide range of applications—from aircraft and aerospace engineering through automotive, robotics and biomedical engineering and up to civil engineering [1,2]. For applications such as aircraft and bridges, one of the most important elements of a smart system is structural health monitoring (SHM) [3,4]. The purpose of performing SHM is to detect structural damage and determine the current state of health of the structure. A typical SHM system consists of three major parts: (1) sensors that are either embedded in the structural elements or attached to their surfaces, (2) cables that transmit the signals from sensors and (3) a data acquisition/processing unit. Correlations exist between smart systems and biological ones; both possess three elementary components: sensors (nerves), a control unit (brain) and actuators (muscles). For a typical biological system, nerves are responsible for detecting external stimuli such as touch and sending corresponding signals to the brain. The brain processes the information received from the nerves and makes decisions about further actions. Subsequently, the brain sends a signal to motor units which each consist of a motor neuron and innervated skeletal muscle fibers. The goal is to move a specific part of the body (structure) by transforming the electrochemical energy into mechanical energy. In analyzing the idea of smart systems by simulating biological systems, one concludes that making such a system functional requires the use of a large number of distributed sensors. However, such an application is not practical with the conventional production and implementation methods. To overcome the stated problem, sensor networks where miniscule sensors and wires are co-fabricated and can be accommodated to a wide variety of shapes and scales are necessary.

With the rapid and flourishing development of flexible electronics, technological barriers to fabricating unconventional compliant and adaptable circuits are being constantly broken. Inspired by the art of Kirigami, Rogers et al. have established experimental and theoretical approaches for fabricating silicon-based highly stretchable micro-circuits that are ultrathin, conformable and can be softly laminated onto the surface of human skin or organs to enable epidermal sensing and surgical therapy monitoring [5,6,7]. Rather than using traditional silicon-based semiconductors, Bao et al. focus on the research of intrinsically stretchable materials serving as the building blocks of functional skin electronics and taking advantages of polymeric properties such as self-healing and biodegradability to better interface with humans [8,9,10]. With their excellent mechanical and electronic properties, carbon nanotubes are another popular candidate for forming flexible and stretchable circuitry; Javey et al. utilized solution-processed nanotube networks integrated with various organic and inorganic materials over large-areas on a single plastic substrate to create a user-interactive electronic skin with instantaneous pressure visualization capability [11,12]. Additionally, the emergence of 3D printed electronics provides great opportunities for the mass production of flexible electronics owing to its low-cost facile process. Enabled by these printing technologies, a wide range of human-machine interface applications have been developed by Arias et al. in wearable electronics, interactive displays, sensor arrays, energy storage/harvesting devices and so forth. [13,14]. Stretchability is differentiated from flexibility by in-plane deformation and out-of-plane deformation conditions. While other components can remain unchanged, a typical change from flexible electronics to stretchable electronics is in the substrate and the interconnections, which must be made stretchable rather than flexible. Most of the aforementioned work represents typical “system-on-plastic” demonstration of flexible electronics and relatively little of it reflects the narrow definition of “stretchable electronics.” In fact, in a restricted concept of stretchability, researchers generally refer it as the “stretch” of materials, which inevitably results in undesired changes in material properties when the material is stretched to some extent. Thus, the stretchability is invariably small, usually less than 200% and the stretching is always limited by the least stretchable part of the system.

**Figure 1:**
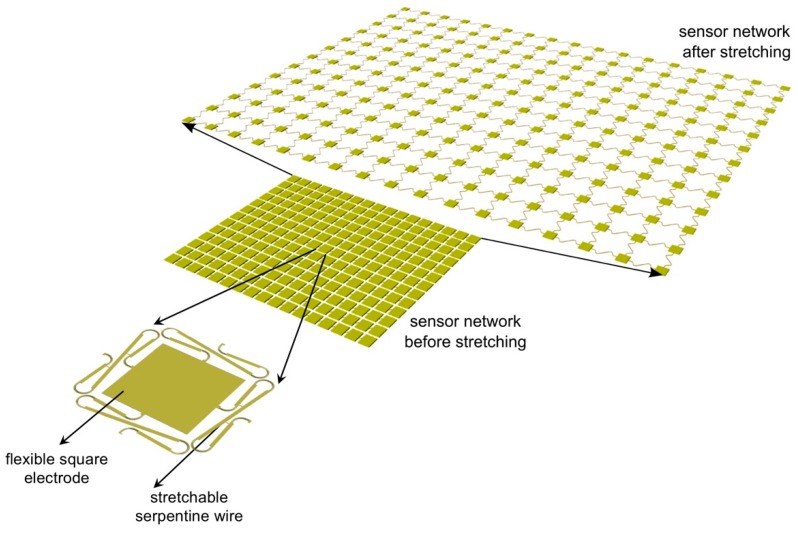
The stretchable sensor network concept.

To extend the concept of stretchability to a greater scale, we would like to revolutionize the definition of stretchable platform by replacing the idea of stretching the materials with the idea of stretching the structures. After our research group first introduced a highly expandable sensor network [15], there has been tremendous effort in the literature to put forth the concept of stretchable network platforms that serve as interconnections for built-in rigid island sensor nodes [16,17,18,19,20,21,22]. Stretchable sensor networks take advantage of a stretchable serpentine wire structure as shown in Figure 1 to connect flexible square electrodes to form a network structure. The curved section of the serpentine wire is intentionally designed to be split to minimize stress concentration and out-of-plane deformation. Different combinations of the number and the length of the straight section of the serpentine wire offer various stretchabilities to the stretchable network. The flexible square electrodes have two purposes; some of them can be integrated with sensors, such as resistive temperature detectors (RTD) and piezoelectric transducers (PZT), while some of them only act as the dummy nodes to provide electrical connection. All the signals generated from the sensor nodes are routed through the dummy nodes to the periphery of the network so that a flexible printed circuit board can be attached by soldering the outmost bond pads to transmit the signals to a signal conditioning circuit where they are collected using a data acquisition system. In practice, stretchable sensor networks have proven to be effective in many applications. As an example, Guo et al. developed a stretchable sensor network with 8 PZTs and 23 RTDs to act as an artificial electronic skin for robots [18]. After a 700% area increase, the network was deployed on a robotic arm to detect heat and impact. A feedback control algorithm was also implemented to enable automatic control of the robotic arm in response to identified stimuli. Additionally, by embedding 4 of these sensor networks inside a prototype composite wing, Kopsaftopoulos et al. developed a stochastic global identification framework to allow real-time state awareness of aerospace structures operating under varying flight states [21].

Although these stretchable sensor networks have demonstrated the functionality of sensing various parameters and adaptability to different structures, strain sensing is still one of the remaining missing pieces of the puzzle. Hence, the objective of this work is to integrate robust strain measurement capability to these sensor nodes. Strain measurements can be performed using non-piezo resistive modalities such as fiber-optic sensors [23,24] and piezoelectric sensors [25,26]. However, the prevailing transduction mechanism is piezo resistive. Numerous papers have discussed piezo resistive based strain sensing by introducing advanced nanomaterials including carbon nanotubes [27], silver nanowires [28] and graphene [29]. Although these strain sensors have demonstrated high tolerable strains and gauge factors of a single unit under macroscopic strains, they cannot be integrated to the stretchable sensor network platform for three main reasons: (1) they are not compatible with the microfabrication process, thus cannot be mass produced with a high yield; (2) they cannot be built at a relatively small size, thus limiting their spatial resolutions; (3) they have not yet been proven to be robust and reliable in fatigue tests. Metal foil strain gauge technology is well-known for its linearity and stability. The simple structure of metallic wires also makes it fabrication-friendly compared to semiconductor strain gauges [30]. The principle of metal foil strain gauges is straightforward. As force is applied, the sensing element (i.e., metallic resistive foil) undergoes elastic deformation, changing its resistance value and generating an electrical output signal via a bridge circuit. Unfortunately, resistance is also a function of temperature; hence to minimize such thermal effect, novel temperature compensation techniques have been tried by researchers including creating alternative sensor design [31] and tuning applied biased voltage [32]. Building upon the existing stretchable network platform, this paper aims to add additional distributed strain sensing capability by designing and fabricating minuscule metal foil strain gauges on the sensor nodes. The network is then stretched and laminated to an aluminum sheet to provide it with real-time strain sensing functions. To further validate the performance of the strain gauges, tensile tests, fatigue tests and flexural tests have been performed to fully characterize their linearity, sensitivity and reliability.

## 2. Problem Statement

The goal of this work is to design a strain gauge pattern that fits the sensor network platform and can be mass produced at a low cost and a high yield using microfabrication processes. The resulting strain gauge network should have low tolerance, high reliability and should be deployable over large structural areas and in various configurations to provide real-time strain sensing capabilities.

## 3. Method of Approach

The main objective of this work is to develop and characterize a network of strain gauges. To achieve this goal, the method of approach can be divided into three major tasks: design, fabrication and testing. The design part includes material selection, strain gauge sensor node design and the design of the network platform from the standpoint of optimizing the strain gauge performance. The fabrication part includes the microfabrication of the stretchable sensor network, network stretching and structural integration. The testing part includes the tensile test for calibrating gauge factors, the fatigue test for validating sensor reliability and robustness and the flexural test for demonstrating strain distribution capability.

## 4. Sensor Network

### 4.1. Design and Simulation

**Figure 2:**
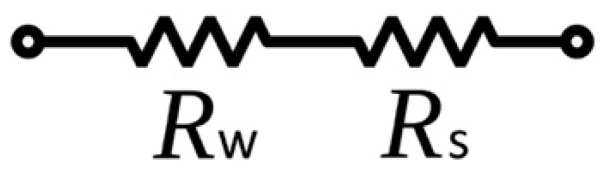
Circuit diagram of stretchable wire and strain gauge.

Since the metal foil strain gauge is a resistor-based sensor that functions by measuring a relative change in resistance upon structural deformation, the appropriate design principle is to determine the optimum resistance value and the corresponding resistor geometry. As the stretchable wire and the strain gauges are connected in series (Figure 2), the first design requirement is to minimize the resistance contribution from the stretchable wire (Rw), by increasing the resistance values of the strain gauges (Rs). Thermal noise, which is proportional to the square root of resistance, should be reduced, so Rs must be limited. A reasonable 1% ratio of Rw to Rs has been determined by considering both factors and comparing these with those corresponding to commercially available strain gauges [33]. Each serpentine structure of a stretchable wire has a resistance value of 18 Ω. By multiplying the number of serpentines, Rw ranges from 72 to 396 Ω with an average value of 116 Ω. Thus, the resistances of the strain gauges are designed to be about 11.6 kΩ. Constantan is selected for fabricating strain gauges as it is the material of choice for most commercial strain gauges owing to its high resistivity, sensitivity and low temperature coefficient. After determining the resistances and materials of the strain gauge, the geometry parameters can be derived using Table 1.

**Figure 3:**
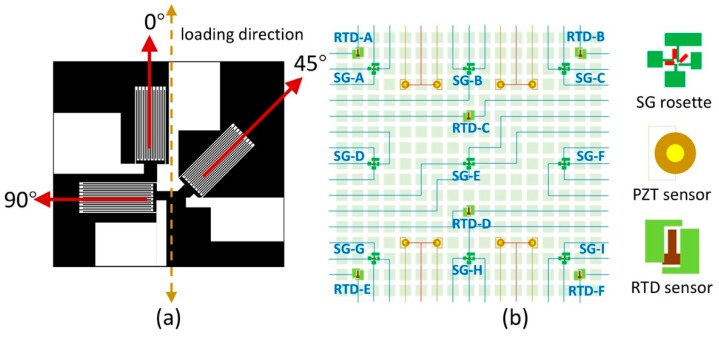
(**a**) Sensor node design of strain gauge rosette in 0°, 45°, 90°; (**b**) Sensor layout and wiring diagram of a 17 × 17 network with 9 strain gauge (SG) rosettes, 8 piezoelectric transducers (PZT) and 6 resistance temperature detectors (RTD) distributed centrosymmetrically.

**Figure 4:**
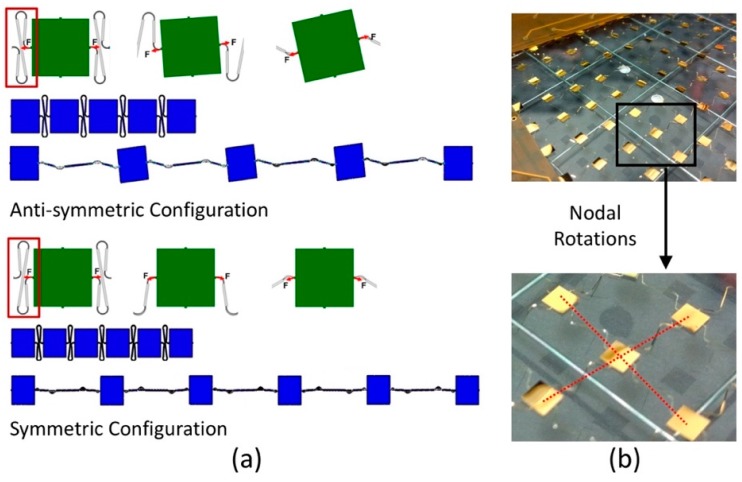
(**a**) Finite element modeling of the stretching process is performed to analyze the stress distribution along node-wire connections in two configurations to minimize nodal rotation effect; (**b**) Stretchable sensor networks tend to have node rotations after being stretched.

The strain gauge rosette layout shown in Figure 3a has been designed for in-plane strain applications using two main design elements [34]. Firstly, by using a group of strain gauges in certain angles ($\theta_{a}$, $\theta_{b}$, $\theta_{c}$), we are capable of deriving both the shear strain ($\gamma_{\mathrm{xy}}$) and normal strains ($\varepsilon_{x},\varepsilon_{y}$) from the strain transformation matrix (1) and the measured strains ($\varepsilon_{a}$, $\varepsilon_{b}$, $\varepsilon_{c}$) in three directions on the same plane of a Cartesian coordinate system. As an example, illustrated in Figure 3a, letting $\theta_{a}=0$°, $\theta_{b}=90$°, $\theta_{c}=45$° and assuming the x axis is aligned with the loading direction, then $\varepsilon_{x}=\varepsilon_{a}$, $\varepsilon_{y}=\varepsilon_{b}$, $\gamma_{\mathrm{xy}}=2\varepsilon_{c}-\left(\varepsilon_{a}+\varepsilon_{b}\right)$. Secondly, by using a half-bridge in the form of a 0–90 degree Tee rosette and aligning one gauge to the principle strain direction, the strain gauge can be used for a uniaxial strain measurement with excellent temperature compensation when transverse strains act according to the Poisson’s ratio and no steep temperature gradient exists. However, to exert the power of strain gauge rosettes, precise alignment is critical. It was observed in previous work that the stretchable sensor networks tend to have node rotations after being stretched (Figure 4b). This is irrelevant when measuring non-directional scalar quantities such as temperature but important when performing strain/stress measurements. Therefore, we simulated the stretching process of the wires and analyzed the forces on the nodes in two different node-wire configurations using finite element methods, which can be seen in Figure 4a. In the centrosymmetric configuration, the torque caused by a pair of mismatched forces leads to the rotation of the sensor node. In contrast, when the two wires are axisymmetrically configured, the two counter-acting forces balance each other to avoid nodal rotations.

(1) $$\;\left(\begin{array}{c} \varepsilon_{x}\\ \varepsilon_{y}\\ \gamma_{\mathrm{xy}} \end{array}\right)={\left[\begin{array}{ccc} \cos^{2}\theta_{a} & \sin^{2}\theta_{a} & {\sin\theta }_{a}{\cos\theta }_{a}\\ \cos^{2}\theta_{b} & \sin^{2}\theta_{b} & {\sin\theta }_{b}{\cos\theta }_{b}\\ \cos^{2}\theta_{c} & \sin^{2}\theta_{c} & {\sin\theta }_{c}{\cos\theta }_{c} \end{array}\right]}^{-1}\left(\begin{array}{c} \varepsilon_{a}\\ \varepsilon_{b}\\ \varepsilon_{c} \end{array}\right)\;$$

The locations of particular sensors post-stretch can be predetermined by simulating the effects of external stimulus on target materials appropriate to the design application. Based on previous work, we redesigned the stretchable sensor network developed by Guo and added strain gauges to it [18]. First, a 3 × 3 strain gauge (SG) rosette matrix was evenly spread out to cover the entire network. Then, 4 RTDs were assigned to 4 corners to capture the temperatures associated with the strains while the other 2 RTDs located along the center line were assigned to sense the thermal gradient in the direction where the sensor network is oriented. Finally, 4 pairs of PZT electrodes were placed accordingly to monitor vibrations and mechanical impacts. Since PZTs are stiff and brittle, they are not suited for curved surfaces, which limits their design flexibility. To fit PZTs in the stretchable sensor network, we selected the smallest piece of piezo plate to have dimensions of 2 × 2 × 0.2 mm. Previous work has demonstrated that PZTs can be directly mounted on a pipeline with similar radius of curvature [35], thus the curvature limitations of the network with PZTs is 0.5 mm^−1^. The demonstration of such PZT network-based structural health monitoring systems can be found elsewhere in References [16,17] and is not within the scope of this study. In total, a single network contains 27 SGs, 6 RTDs and 8 PZTs. The SGs and RTDs were named alphabetically according to left-to-right and top-to-bottom sequence (Figure 3b). Each SG of a rosette was specifically labeled with respect to its angle to the vertical (0, 45, 90).

### 4.2. Sample Preparation

#### 4.2.1. Sensor Network Fabrication

**Figure 5:**
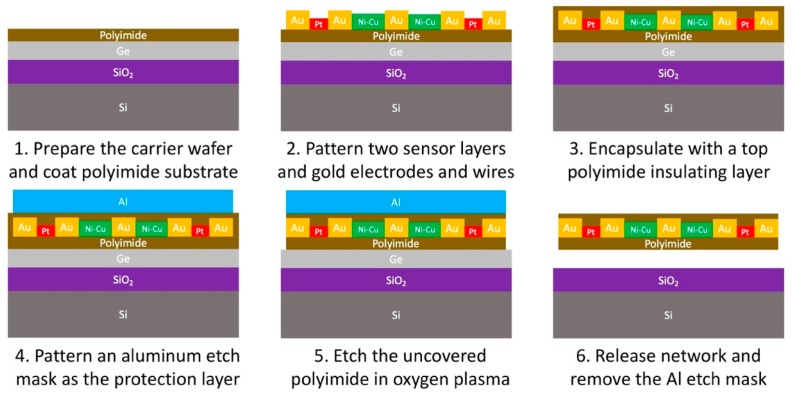
Fabrication process flow of the multifunctional stretchable sensor network.

The entire microfabrication process was completed in the cleanroom of the Stanford Nanofabrication Facility. A schematic diagram illustrating the process flow is shown in Figure 5. More details can be found by referring to our previous work [18]. The process begins with thermally growing a 1.5 μm silicon dioxide etch stop layer on a supporting silicon wafer through LPCVD, then depositing a sacrificial layer of chromium/germanium with 10/300 nm thickness by e-beam evaporation in vacuum. Two layers of PI-2611 polyimide with a thickness of about 14 μm and one layer of the mixture of polyimide PI-2545 and thinner T-9039 with a ratio of 1:2 and a thickness of 1 μm are then spin-coated onto the prepared carrier wafer as the substrate of the stretchable network. Constantan alloy, platinum and gold layers are deposited using e-beam evaporator and patterned with photolithography to create the strain gauges, temperature sensors and stretchable wires, respectively. A thin encapsulation of the PI-2545/T-9039 mixture is then formed using spin coating technique and serves as the insulating and protection layer. Then, a 40/700 nm titanium/aluminum etch mask is patterned with photolithography, metallization and lift-off to cover all the nodes and wires. In the subsequent step, oxygen plasma is applied to anisotropically etch away any unprotected polyimide. Finally, the network is released from the supporting wafer by dry etching the germanium sacrificial layer with xenon difluoride followed by removing the aluminum etch mask in diluted hydrofluoric acid.

#### 4.2.2. Sensor Network Deployment and Integration

**Figure 6:**
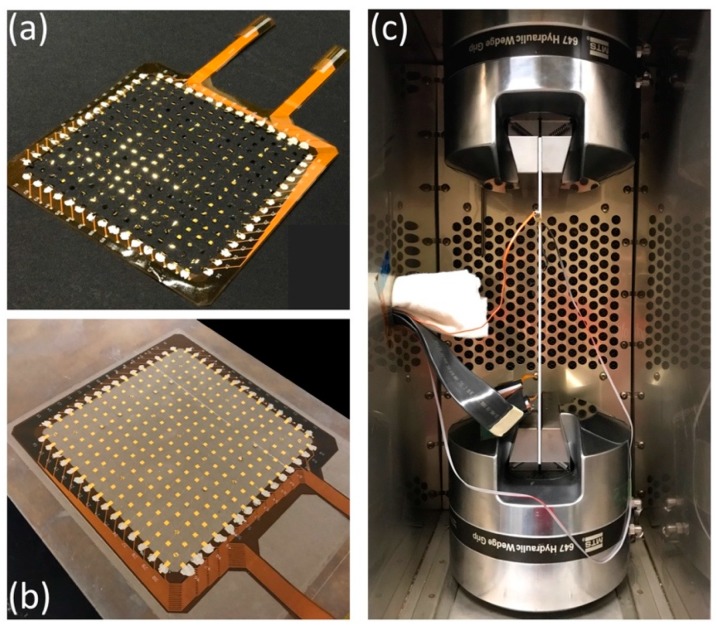
(**a**) Sensor network installed to a customized flexible printed circuit board for signal transmission; (**b**) Sensor network integrated to an aluminum sheet using vacuum bagging technique; (**c**) Experimental setup of the tensile test performed in an environmental chamber.

The bond pads on the four edges of the square network were intentionally connected in order to hold the flexible sheet in shape and increase the stability of the network during the release process. To stretch the network in one direction, the connections between bond pads were cut using a blade and then the edges that would not be stretched were gently attached to a pair of fixtures. The stretching process was done by manually pulling away the two fixtures in a constant speed. Care was taken to not stretch the network fully, as this can cause undesired plastic deformation of the wires. To stretch the network in the other direction, the process was repeated in the remaining direction by carefully transitioning from the first pair of fixtures to a second transverse pair. Next, the stretched network was carefully connected to an in-house designed flexible printed circuit board (PCB) by applying silver paint (Ted Pella, Leitsilber 200, silver content 45%) for electrical conduction (Figure 6a). The silver paint can be dried within 10 min at 20 °C and can sustain temperatures up to 120 °C. The PZTs (piezo plate, 2 × 2 × 0.2 mm, APC) were then surface-mounted to the predesigned electrodes of the network by gluing one electrode to the top and the other to the bottom of the ceramic disk with the same silver paint.

The network was installed onto an aluminum sheet (7075-T6, McMaster-Carr, Elmhurst, IL, USA) with dimensions of 16″ × 8″ × 1/8″ by sandwiching the sensor network between layers of fiberglass and epoxy (Figure 6b). First, the aluminum sheet was cleaned and epoxy precursors (LAM-125, LAM-226, PRO-SET Epoxies, Bay City, MI, USA) were mixed using a 3.5:1 ratio. The epoxy was applied to the aluminum sheet and a fiberglass veil with 150 μm thickness was laid on top of the epoxy. A squeegee was used to gently scrape away excess epoxy and level the surface. The sensor network was then slowly placed over the wet fiberglass veil and aligned with the aluminum sheet. To ensure the fiberglass insulated the sensor network from the aluminum sheet, we used a digital multimeter to perform continuity testing between the two layers and no electrical connections were found. The next step is to put a perforated release film and bleeder above the fiberglass veil to trap and hold the excess epoxy away from the laminate. Then a vacuum-bagging film was used to wrap the entire laminated structure. The laminate was vacuum cured at 82 °C for 6 h in an oven. After curing the epoxy, the extra materials were removed, leaving a thin layer of epoxy covering the sensor network uniformly and conformally without any additional protection layer. Here the vacuum bagging technique ensures the close and strong attachment of the sensor network to the aluminum sheet so that the deformation of the aluminum sheet can be directly transferred to the sensor network. Additionally, a commercial strain gauge (KFH-6-350-C1-11L3M3R, OMEGA Engineering, Norwalk, CT, USA) was placed on the back of the aluminum sheet and was centered and aligned with the long edge of the aluminum sheet in order to provide accurate local strain information. Finally, we soldered the joints of the flexible PCB and used two shielded ribbon cables for signal transmission.

### 4.3. Testing

#### 4.3.1. Analyses of Yield and Variation

**Figure 7:**
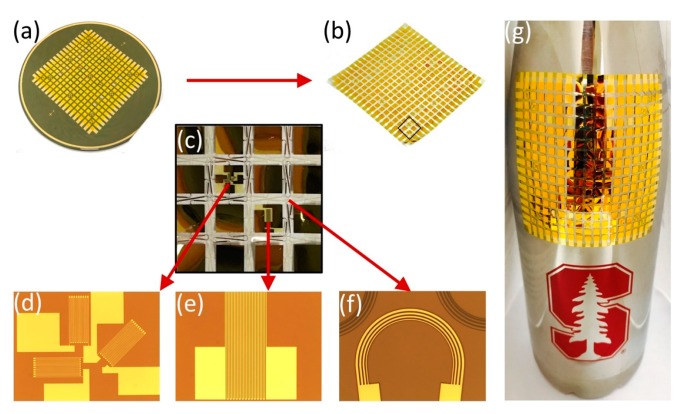
(**a**,**b**) Network appearance before and after release from the supporting wafer; (**c**–**f**) Network details from macro to micro scale; (**g**) A flexible network wrapping an irregularly shaped water bottle.

Figure 7a,b demonstrates the appearance of the microfabricated network before and after the releasing process. Figure 7c–f depicts the network details from the macroscale to the microscale. Figure 7d–f are the microscopic images of the microfabricated strain gauge, RTD and stretchable wire, respectively. As shown in Figure 7g, the sensor network was placed onto a water bottle with moisture on its surface. The capillary stiction forms a close and conformal coating of the sensor network. The water bottle has an irregular shape with changing curvatures (κ_min_ = 26.25 m^−1^, κ_max_ = 28.63 m^−1^), proving the flexibility of the sensor network in adapting to various structures. Two key parameters evaluating the microfabrication process are the yield and variation. Creating a single device is relatively easy but replicating it to form a network structure is much more challenging. Despite the difficulties encountered in the microfabrication process, we managed to realize a 25/27 yield for SGs and a 6/6 yield for RTDs. An uncertainty analysis of the sensor resistance values has been conducted based on the electrical resistance formula for a wire (2), which depends upon the material resistivity (ρ) and geometric parameters (L: length, W: width, T: thickness). The relative uncertainty ($\omega_{R}/R$) (4) can be derived from the uncertainty (ω) formula for product functions (3) [36]. The uncertainty values and their corresponding rationales are given in Table 2 for reference. The calculated relative uncertainty is 3.03% which can be regarded as an estimate of the coefficient of variation (CV), while the actual CV of the resistance values are 2.82% for RTDs and 2.56% for SGs, given by the ratio of the standard deviation to the average. Noting that the networks were fabricated in a Class 100 cleanroom for academic purposes, higher yields and lower variations should be expected in an industry-level cleanroom.

(2) $$\;R=\rho \frac{L}{\mathrm{WT}}.\;$$

(3) $$\;\omega_{R}^{2}={\left(\frac{\partial R}{\partial L}\right)}^{2}\omega_{L}^{2}+{\left(\frac{\partial R}{\partial W}\right)}^{2}\omega_{W}^{2}+{\left(\frac{\partial R}{\partial T}\right)}^{2}\omega_{T}^{2}\;$$

(4) $$\;{\left(\frac{\omega_{R}}{R}\right)}^{2}={\left(\frac{\omega_{L}}{L}\right)}^{2}+{\left(-\frac{\omega_{W}}{W}\right)}^{2}+{\left(-\frac{\omega_{T}}{T}\right)}^{2}\;$$

The stretching process expanded the network from 60 × 60 mm to 150 × 150 mm and preserved it without losing any sensors and wires. The polyimide encapsulation layer introduced during the microfabrication process insulates the sensor network. The epoxy coating resulting from the vacuum bagging installation further prevents the sensor network from any scratches or contamination.

#### 4.3.2. Tensile Test

**Figure 8:**
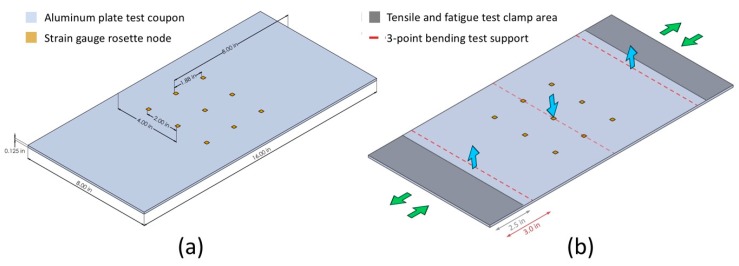
(**a**) Sketches of the test coupon with (**a**) basic dimensions; (**b**) testing dimensions.

**Figure 9:**
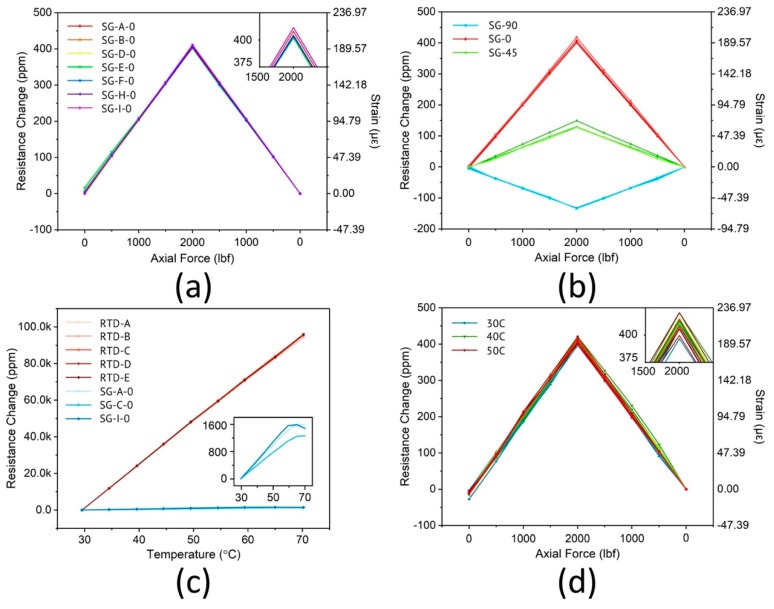
Relative resistance change of the strain gauges in response to load change and the corresponding strains for (**a**) 7 strain gauges in principle stress direction; (**b**) 3 strain gauge rosettes; (**c**) Relative resistance change of the RTDs and strain gauges from 30 °C to 70 °C; (**d**) Relative resistance change of the strain gauges in response to both load and temperature change and the corresponding strains for 5 strain gauges in principle stress direction.

The strain gauges were calibrated by performing tensile testing on the aluminum sheet with a servo-hydraulic test system (MTS Systems Corporation, Model Number: 370.25). First, the rosettes were oriented in such a way that the center strain gauge was parallel to the loading direction as shown in Figure 3a. The positions of the 9 strain gauge rosettes on the aluminum test coupon are illustrated in Figure 8a. The strain gauges were electrically connected to a multi-channel digital multimeter (DMM). The DMM (Keithley Instruments, Model 3706A) has a 7 ½ digit accuracy to record miniscule resistance changes (e.g., 1 mΩ change of a 10 kΩ resistor). The switching frequency of the DMM is limited to 2 Hz, thus dynamic strain measurement is beyond its capacity. The aluminum coupon was loaded and unloaded quasi-statically from 0 to 2000 lbf (Figure 8b), during which data were taken. Figure 9a shows the relative resistance change of the strain gauges in response to the load change. The linear behavior of the strain gauge resistances indicates good consistency corresponding to various load conditions. The linearity is quantified by calculating the R-squared value, which is larger than 0.99 for all trendlines.

A fundamental parameter of the strain gauge is its sensitivity to strain, expressed quantitatively as the gauge factor (GF). GF is the ratio of the fractional change in electrical resistance to the fractional change in physical length. As opposed to other measured quantities, gauge factors are inherent properties depending on the as-fabricated material properties. By using the commercial strain gauge for calibration, the gauge factor of the fabricated strain gauges is 2.11 ± 1.95% Ω/(Ω·ε), versus 2.04 ± 1.0% Ω/(Ω·ε) for the commercial strain gauges. Comparing the percentage variation in the GF, a high degree of uniformity is achieved from the microfabrication process, making the microfabricated strain gauges competitive against the commercial ones. The effect of the wire strain leading to measurement error has also been quantified with the defining equation of the gauge factor (5) and the GF of gold (GF_Au_ = 2.6) [37]. If the sensor has a strain of x and the wire has a strain of y, then the measured strain would be 0.99x + 0.0123y (6). If the strain distribution is uniform, which means x = y, then the error is 0.23%; if the strain distribution is not uniform, when 2x = y, the error is 1.46%; when 3x = y, the error is 2.69%. Compared to the variation in the sensitivity of the gauge, these errors are close but not significantly larger. One can include this factor in the calculation of the nominal values of the GF to minimize the error. The error itself can also be reduced by further increasing the resistance ratio of the sensor to the wire. The strain data recorded from the gauge rosettes were used to calculate the Poisson’s ratio of the aluminum plate (Figure 9b). The Poisson’s ratio is calculated by dividing the strain of transversal compression by the strain of longitudinal elongation; both of these values are measured on the same rosette. The strain that is perpendicular to the tensile force can be directly measured by the 90° strain gauge of the rosette. From the three strain gauge rosettes that were measured, the mean value of the Poisson’s ratio is 0.3306 ± 2.05%, which closely matches the nominal value 0.33.

(5) $$\;\mathrm{GF}=\frac{\Delta R/R}{\varepsilon }\;$$

(6) $$\begin{array}{ll} \;\varepsilon & =\frac{\Delta R/R}{\mathrm{GF}}=\frac{\left(\Delta R_{s}+\Delta R_{w}\right)/R}{\mathrm{GF}}=\frac{\left(x\cdot \mathrm{GF}\cdot R_{s}+y\cdot {\mathrm{GF}}_{\mathrm{Au}}\cdot R_{w}\right)/R}{\mathrm{GF}}\\ & \;=\frac{x\cdot \mathrm{GF}\cdot R_{s}/R+y\cdot {\mathrm{GF}}_{\mathrm{Au}}\cdot R_{w}/R}{\mathrm{GF}}=\frac{x\cdot 2.11\cdot 0.99+y\cdot 2.6\cdot 0.01}{2.11}\\ & \;=0.99x+0.0123y\; \end{array}$$

#### 4.3.3. Temperature Effect

Temperature effects were also studied by characterizing RTDs and SGs simultaneously. The aluminum plate with the sensor network was set in an oven (Cascade TEK) and a commercial thermocouple temperature sensor (5TC-TT-K-24-36, OMEGA Engineering) was attached to the plate for calibration. In the experiments, temperature was increased from 30 °C to 70 °C quasi-statically. As shown in Figure 9c, all 5 RTDs exhibited excellent linear response to temperature change with average R-squared value greater than 0.999. The sensitivity of the RTDs, usually known as the temperature coefficient of resistance (denoted by α in units of Ω/(Ω·°C)) can be calculated from the slope of the resulting curves. Here α = 2.354 × 10^−3^ ± 0.71% Ω/(Ω·°C) in the 30 to 70 °C range. On the other hand, the resistive response of the strain gauges to temperature change is much less significant: only 1.5% of that of the RTDs at the same temperatures. α = 4.591 × 10^−5^ ± 2.01% Ω/(Ω·°C) is calculated for the strain gauges and can be used for temperature compensation. Another experiment was performed with the aim of combining both load and temperature effects. The loading condition was kept the same while an environmental chamber was used to set the temperature at 30, 40 and 50 °C. The experimental setup is shown in Figure 6c. After compensating for the temperature coefficient, the results can be seen in Figure 9d. Most of the curves overlap with each other, demonstrating a good measurement consistency over a wide temperature range.

#### 4.3.4. Flexural Test

**Figure 10:**
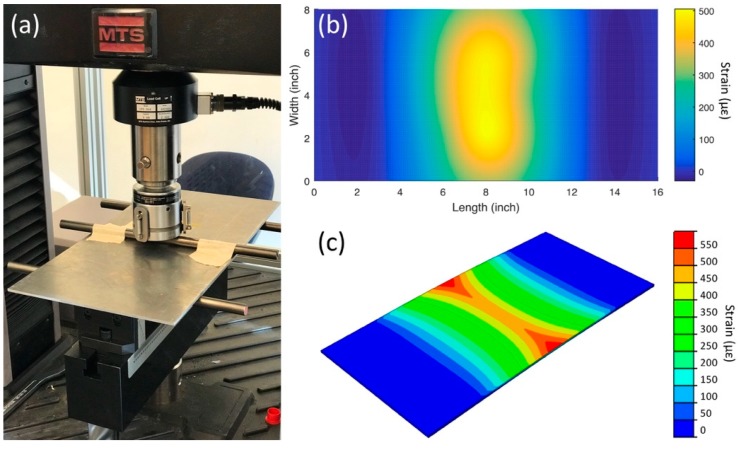
(**a**) Experimental setup of the 3-point bending test; (**b**) Contour plot of the measured strains upon load increments to the coupon; (**c**) Simulated strain profile with a 50 lbf bending force.

To demonstrate the distributed strain measurement capability of the sensor network, a three-point bending flexural test was conducted on the aluminum plate with an electromechanical universal test system (MTS Criterion Model 43). Three-point bending introduces a linear symmetric strain distribution along the longitudinal direction of the aluminum plate with a span length of 10″. (Figure 10a). The experiments were carried out by applying 50 lbf, 100 lbf and 150 lbf transverse loadings at the middle of the coupon while supporting two transverse lines 5″ away from the center (Figure 8b). Strain distribution on the bottom surface of the plate was calculated by interpolating longitudinal strain values read by 9 individual strain gauges in the network. The readings were drawn into a contour plot as seen in Figure 10b. Interpolation was performed using the biharmonic spline interpolation method in MATLAB R2016b. According to the interpolation method utilized, a 2D biharmonic function is determined by solving the biharmonic equation with data points being the individual strain readings and zero-strain boundary conditions at the two intermediate supports and free-ends [38]. A finite element simulation of the three-point bending test was performed in ABAQUS to correlate with the experiments. The model was built with three-dimensional solid C3D8R elements. Simply-supported boundary conditions were reproduced by restraining vertical translation of the nodes at the two free edges and two intermediate supports. Loading was simulated by applying 50 lbf force through the centerline in the transverse direction of the plate. The simulated strain profile can be seen in Figure 10c. Comparing the strain values along the centerline in longitudinal direction of the plate, the simulation closely matches the experiment with a difference of 3.59%.

#### 4.3.5. Fatigue Test

To demonstrate the reliability of the sensor network, we performed a fatigue test on the aluminum plate with the MTS machine. Sinusoidal cyclic loads with 0.2 Hz cycling frequency, 3000 psi maximum stress and 0 psi minimum stress were applied to the specimen. This stress and strain level corresponds to 5% of the yield strength (61,000 psi) of the 7075 aluminum alloy and 3% of the ultimate elongation of the constantan alloy [39]. To verify that the cyclic loads yield the same level of strain without any delay, we monitored 10 cycles by collecting the strain data from 12 strain gauges on the network at a scan rate of 2 Hz and compared the maximum and minimum values with the static load conditions. After the verification, the test was started with 2500 cycles, followed by a quasi-static tensile test, which is identical with the previous calibration experiment. The same test was repeated 5 times. After 12,500 cycles in total, all the 12 tested strain gauges behaved consistently as before, proving the sensor network has desirable reliability and practicability for SHM applications.

## 5. Conclusions

In summary, a stretchable strain gauge network has been designed, fabricated and integrated with a yield over 90% and a variation below 3%. The strain gauges have been fully calibrated and characterized in terms of linearity, sensitivity and temperature coefficient. The stretchable network can be expanded 625% to cover a large area and the sensor readings are not sensitive to any non-uniform deformation of the target structure. The network has a size ranging from 60 × 60 mm to 150 × 150 mm (depending on the degree of stretch) and can be operated at 300 micro-strain for more than 10,000 cycles. The strain gauge network is integrable with common structures by vacuum bagging techniques, capable of rendering a distributed strain/temperature profile and is reliable for damage detection in machinery, aerospace and civil structures.

## Figures and Tables

**Table 1:** Geometric Parameters and Resistance Values of Microfabricated Strain Gauges (SG).

	Length	Width	Thickness	Number of Strips	Theoretical Resistance	Actual Resistance
SG	500 µm	4 µm	100 nm	18	11.025 kΩ	11.831 kΩ

**Table 2:** Uncertainty Values and Corresponding Rationales.

Uncertainty	Value	Rationale
$\omega_{L}$	± 2 µm	photolithographical alignment accuracy
$\omega_{W}$	± 2 µm	
$\omega_{T}$	± 4 nm	metal deposition empirical data
